# 12/15-Lipoxygenase Contributes to Platelet-derived Growth Factor-induced Activation of Signal Transducer and Activator of Transcription 3*

**DOI:** 10.1074/jbc.M113.489013

**Published:** 2013-10-28

**Authors:** Tina Blažević, Andrea V. Schwaiberger, Cornelia E. Schreiner, Daniel Schachner, Anja M. Schaible, Christoph S. Grojer, Atanas G. Atanasov, Oliver Werz, Verena M. Dirsch, Elke H. Heiss

**Affiliations:** From the ‡ Department for Pharmacognosy, University of Vienna, Althanstrasse 14, A-1090 Vienna, Austria and; the § Chair for Pharmaceutical/Medicinal Chemistry, University of Jena, Philosophenweg 14, D07743 Jena, Germany

**Keywords:** Chemical Biology, Lipoxygenase Pathway, Redox Regulation, Signal Transduction, STAT3, Vascular Smooth Muscle Cells, Indirubin-3′-monoxime, Platelet-derived Growth Factor

## Abstract

We showed previously that the small molecule indirubin-3′-monoxime (I3MO) prevents vascular smooth muscle cell (VSMC) proliferation by selectively inhibiting signal transducer and activator of transcription 3 (STAT3). Looking for the underlying upstream molecular mechanism, we here reveal the important role of reactive oxygen species (ROS) for PDGF-induced STAT3 activation in VSMC. We show that neither NADPH-dependent oxidases (Noxes) nor mitochondria, but rather 12/15-lipoxygenase (12/15-LO) are pivotal ROS sources involved in the redox-regulated signal transduction from PDGFR to STAT3. Accordingly, pharmacological and genetic interference with 12/15-LO activity selectively inhibited PDGF-induced Src activation and STAT3 phosphorylation. I3MO is able to blunt PDGF-induced ROS and 15(*S*)-hydroxyeicosatetraenoic acid (15(*S*)-HETE) production, indicating an inhibitory action of I3MO on 12/15-LO and consequently on STAT3. We identify 12/15-LO as a hitherto unrecognized signaling hub in PDGF-triggered STAT3 activation and show for the first time a negative impact of I3MO on 12/15-LO.

## Teaser

**Background:** The small molecule indirubin-3′-monoxime (I3MO) inhibits activation of STAT3 in vascular smooth muscle cells, with an unresolved mechanism.

**Results:** Activation of 12/15-lipoxygenase (LO) is crucial for PDGF-induced Src and STAT3 activation and is impaired by I3MO.

**Conclusion:** I3MO interferes with PDGFR-Src-STAT3 signaling via impaired 12/15-LO activation.

**Significance:** 12/15-LO is an important signaling hub within the PDGF-STAT3 pathway.

## Introduction

Platelet-derived growth factor (PDGF)^2^-induced proliferation of vascular smooth muscle cells (VSMC) substantially contributes to a reduced vessel lumen as found in atherosclerotic plaques or during restenosis after stent placement. We showed previously that the small molecule indirubin-3′-monoxime (I3MO) inhibits VSMC proliferation induced by PDGF *in vitro* and reduces vessel narrowing *in vivo* (1). The antiproliferative effect of I3MO, a derivate of the naturally occurring compound indirubin used in traditional Chinese medicine against cancer (2), was linked with the selective inhibition of signal transducer and activator of transcription 3 (STAT3). STAT3 phosphorylation at Tyr^705^ and activation seem to be pivotal for VSMC proliferation (3, 4). Other PDGF-activated mitogenic kinases, like Akt and ERK1/2, were not influenced by I3MO (1). How I3MO provokes this selective action has, however, not been resolved.

PDGF-mediated STAT3 activation is still incompletely understood. PDGF is a family of five dimeric isoforms: PDGF-AA, -AB, -BB, -CC, and -DD, formed by four genetically different, but structurally related polypeptide chains. They exert their effects on cells through differential binding to two related tyrosine kinase receptors, PDGF receptor α and β, causing their homo- or heterodimerization. PDGF-BB, a ligand isoform used in this study, can trigger signal transduction mediated by all three receptor dimers, PDGF receptor-αα, -αβ, and -ββ (5). PDGF-induced mitogenesis in rat VSMCs is mediated by PDGF receptor-ββ homodimers, because only the β receptor is expressed (6).

PDGF-induced phosphorylation of STAT proteins may be directly mediated by the PDGF receptor tyrosine kinase after recruitment of the Src homology 2 domain-containing STAT proteins to specific receptor autophosphorylation sites (7, 8). However, direct binding to the PDGF receptor has only been proven for the STAT5b isoform (9). A recent paper (10) reveals the important adapter function of Fer kinase for PDGF-induced and Src-mediated STAT3 phosphorylation in fibroblasts. STAT3 was also described as a downstream target of Src kinase in cancer cells (11). One study (12), demonstrated PDGF-induced STAT3 signaling in fibroblasts in the absence of activated Src.

Several publications have described an increased STAT3 phosphorylation and its translocation into the nucleus upon exposure to elevated levels of reactive oxygen species (ROS) (13–17). ROS are crucial for propagating signals from receptor tyrosine kinases, like PDGF- and EGF-receptor (18–20). They comprise a group of oxygen-derived molecules that are formed in different redox processes (21). Several different enzymatic systems act as producers of ROS in VSMCs including NAD(P)H oxidases, mitochondria, and lipoxygenases (22). Their increased expression and activation could be linked to hyperproliferative cardiovascular diseases, like atherosclerosis and restenosis (19, 23). We therefore hypothesized that PDGF-induced STAT3 phosphorylation is ROS-mediated in VSMCs and that STAT3 inhibition by I3MO involves interference with ROS production. Aims of this study were: (i) to reveal whether ROS are crucial for STAT3 phosphorylation in VSMC, (ii) whether PDGF-induced ROS formation was inhibited by I3MO, and if so (iii) the source of ROS responsible and affected by I3MO.

## EXPERIMENTAL PROCEDURES

### 

#### 

##### Materials and Reagents

I3MO was purchased from Enzo Life Sciences (Lausen, Switzerland). PD 146176 and ebselen were from Cayman Chemicals (Tallinn, Estonia), whereas gp91ds-*tat* (RKKRRQRRRCSTRIRRQL-NH_2_), and its scrambled counterpart, gp91ds-scr (RKKRRQRRRAGAGAGAGA-NH_2_), were ordered from Caslo Laboratory (Lyngby, Denmark). Nordihydroguaiaretic acid (NDGA) and diphenyleneiodonium chloride (DPI) were purchased from Sigma. H_2_O_2_ and human recombinant PDGF-BB (in text referred to as PDGF) were ordered from Carl Roth (Karlsruhe, Germany) and Bachem (Weil am Rhein, Germany), respectively. Purified hydroxy- and hydroperoxyeicosatetraenoic acid (15(*S*)-HETE and 15(*S*)-HpETE) were obtained from Biomol (Sanova, Vienna). The antibodies directed against phosphorylated Tyr^579^ or Tyr^857^ of PDGFRβ and against phosphorylated Tyr^416^ of Src were from Life Tech (Vienna, Austria). Anti-actin and anti-mouse horseradish peroxidase-linked secondary antibody was from MP Biomedicals (Eschwege, Germany). All other primary antibodies used in this study as well as the secondary anti-rabbit antibody, were from Cell Signaling (Danvers, MA).

##### Cell Culture

Unless stated otherwise, the experiments presented in this study were performed in VSMCs isolated from thoracic aortas of Sprague-Dawley rats using a digestion method, as described previously (24). Cells were passaged twice a week and cultured in Dulbecco's modified Eagle's medium (DMEM) supplemented with 2 mm l-glutamine, 100 units/ml of penicillin, 100 μg/ml of streptomycin, and 10% newborn calf serum (all from Lonza, Basel, Switzerland) at 37 °C and 5% CO_2_. Passages 5 to 14 were used in this study. Primary human aortic smooth muscle cells (hAOSMCs) were purchased from Lonza. They were passaged twice a week and maintained at 37 °C and 5% CO_2_ in SmBM basal medium supplemented with SmGM®-2 Single Quots® (insulin, hFGF-B, gentamicin/amphotericin B, 5% FBS and hEGF), all from Lonza (Basel, Switzerland). Passages 5 to 12 were used for experiments. The heterozygous and homozygous 12-LO knock-out mouse embryonic fibroblasts (MEFs) were kindly provided by Dr. Marcus Conrad, German Research Center for Environmental Health, Munich, Germany. Cells were passaged twice a week and cultured in DMEM supplemented with 2 mm l-glutamine, 100 units/ml of penicillin, 100 μg/ml of streptomycin, and 10% fetal bovine serum (all from Lonza) at 37 °C and 5% CO_2_. Cells were grown to subconfluence and serum starved for 24 h, if not stated otherwise, in the appropriate medium supplemented with 0.1% serum, before each experiment.

##### Western Blot

Unless stated otherwise, for all Western blot experiments, serum-starved cells were preincubated with compounds for 30 min and subsequently stimulated with PDGF-BB (20 ng/ml), 15(*S*)-H(p)ETE (1 μm), or H_2_O_2_ for the indicated periods of time. Afterward cells were lysed with an ice-cold lysis buffer (50 mm HEPES, 50 mm NaCl, 10 mm DTT, 50 mm NaF, 10 mm Na_4_P_2_O_7_ × 10 H_2_O, 5 mm EDTA, 1 mm Na_3_VO_4_), supplemented with 1 mm phenylmethylsulfonyl fluoride (PMSF), 1× Complete^TM^ (Roche Applied Science), and 1% (v/v) Triton X-100. Lysates were centrifuged at 5600 × *g* at 4 °C for 10 min and supernatants were used for protein denaturation in 3× SDS sample buffer for 8–10 min at 95 °C. Protein concentrations were determined using Rotiquant reagent according to the manufacturer's instructions (Carl Roth). If not stated otherwise, 20 μg of protein was separated by 7.5% polyacrylamide SDS-PAGE. Standard gel electrophoresis and blotting techniques were used (Bio-Rad Laboratories). Proteins were visualized after antibody incubation using an LAS-3000 luminescent image analyzer (Fujifilm, Tokyo, Japan) and quantified with AIDA^TM^ software, version 4.06 (Raytest GmbH, Straubenhardt, Germany). Densitometric values of phosphorylated proteins were always normalized to those of the corresponding total protein. If applicable, those normalized data were expressed as fold-change compared with control cells whose response was set 100%. Stripping of membranes was performed by incubation in 0.5 n NaOH for 10–20 min.

##### Co-immunoprecipitation

VSMC were serum starved and pretreated as indicated for 30 min. Then cells were stimulated with PDGF (20 ng/ml) for 5 min and immediately lysed. Anti-PDFGRβ antibody (1:100 dilution) was added to 750 μg of protein extract and incubated on ice for 3 h. After another 45 min rolling end-over-end with protein-Sepharose A/G beads (Santa Cruz, Heidelberg, Germany) at 4 °C the immunoprecipitates were collected, washed three times with cold lysis buffer, separated from the beads by boiling in SDS sample buffer for 5 min, and subjected to immunoblot analyses as indicated. Specificity of the pulldown was assured in pilot experiments using an isotype control antibody.

##### Src Kinase Assay in Vitro

The nonradioactive Src kinase assay was performed using the tyrosine kinase assay kit (chemiluminescent) according to the manufacturer's instructions and a recombinant Src (human) protein, both from Milipore^TM^ (Billeria, MA). The luminescence was measured 15 min after the addition of the substrate with a Tecan GENios^TM^ Pro Microplate reader (Tecan, Mannersdorf, Switzerland).

##### Determination of Cell-free Activity of Isolated Human Recombinant cPLA_2_α

The cPLA_2_α coding sequence was cloned from pVL1393 plasmid (kindly provided by Dr. Wonhwa Cho, University of Illinois at Chicago) into pFastBac^TM^ HT A containing a His_6_ tag coding sequence. The recombinant plasmid was transformed into DH10Bac^TM^ *Escherichia coli*. Sf9 cells were transfected with recombinant bacmid DNA using Cellfectin® reagent and the generated baculovirus was amplified. His-tagged cPLA_2_ was overexpressed in baculovirus-infected Sf9 cells and isolated using nickel-nitrilotriacetic acid-agarose beads. Multilamelar vesicles were prepared by drying 1-palmitoyl-2-arachidonyl-*sn*-glycero-3-phosphocholine and 1-palmitoyl-2-oleoyl-*sn*-glycerol in a 2:1 ratio (in chloroform) under nitrogen in glass vials. After addition of 20 mm Tris buffer (pH 7.4), 134 mm NaCl and 1 mg/ml of fatty acid-free BSA were added. Multilamelar vesicle suspension was disrupted by several freeze-thaw cycles (liquid nitrogen). The suspension was extruded 11 times with a mini-extruder (Avanti Polar Lipids, Inc.) through a polycarbonate membrane (100 nm pore diameter) at room temperature (above transition temperature of the lipids) to produce large unilamelar vesicles. The final total concentration of lipids was 250 μm in 200 μl. Test compounds and 1 mm Ca^2+^ were added to the vesicles, and the reaction was started by addition of 500 ng of His-tagged cPLA_2_α (in 10 μl of buffer). After 1 h at 37 °C, 1.6 ml of MeOH was added, and arachidonic acid was extracted by RP-18 solid phase extraction. Following derivatization with *p*-anisidinium chloride, the resulting derivate was analyzed by RP-HPLC at 249 nm (25).

##### Intracellular ROS Measurement

Serum-starved cells were covered with HBSS buffer (140 mm NaCl, 5 mm KCl, 0.14 mm Na_2_HPO_4_, 0.37 mm KH_2_PO_4_, 1.2 mm CaCl_2_ × 2H_2_O, 0.8 mm MgSO_4_, 5.5 mm d-glucose, and 20 mm HEPES, pH 7.4), pre-treated with DMSO, I3MO, or DPI for 30 min and stimulated with PDGF-BB for 10 min. 2′,7′-Dihydrodichlorofluorescein-diacetate (H_2_DCF-DA, 20 μm) was added 15 min before PDGF stimulation, and from this step the experiment was performed in the dark. Cells were trypsinized and re-suspended in 2% BSA/PBS solution. The fluorescence was measured using the flow cytometer FACS Calibur^TM^ (BD Biosciences) at the excitation/emission wavelength of 488/530 nm.

##### Extracellular H_2_O_2_ Measurement

Serum-starved VSMCs were treated with either DMSO or I3MO for 30 min, the medium was aspirated, and the cells were washed once with PBS. They were then covered with KRPG buffer (145 mm NaCl, 5.7 mm Na_2_HPO_4_, 4.86 mm KCl, 0.54 mm CaCl_2_ × 2H_2_O, 1.22 mm MgSO_4_ × 7H_2_O, 5 mm glucose), supplemented with 0.01 mmol/liter of Amplex® Red reagent and horseradish peroxidase, 1 unit/μl (both from Invitrogen), and equilibrated for 5 min at 37 °C and 5% CO_2_. Cells were subsequently stimulated with PDGF-BB (20 ng/ml) in the presence or absence of 150 units of catalase (Sigma) for 30 min. 80 μl of supernatants, in triplicate for each treatment condition, were transferred into a black 96-well plate (PS-Microplate, transparent, flat bottom, Greiner Bio-One GmbH, Frickenheim, Germany) and fluorescence intensity was measured at 535/590 nm (excitation/emission) in the plate reader Tecan GENios^TM^ Pro (Tecan, Mannersdorf, Switzerland). Fluorescence values from wells containing catalase were subtracted from those without catalase to obtain the values for H_2_O_2_ specific fluorescence. External addition of H_2_O_2_ (0.5 μm) to one set of cells served as a positive control in the experiment.

##### 15(S)-HETE Enzyme Immunoassay

Cells were seeded in 10-cm dishes, grown to subconfluence, and serum-starved for 24 h. They were treated with vehicle or compounds for 30 min and stimulated with PDGF-BB for 10 min. Eicosanoid extractions out of supernatants were performed using HyperSep C18 SPE columns (200 mg/3 ml, Thermo Scientific, Runcorn, UK) as instructed in the manual of the 15(*S*)-HETE enzyme immunoassay kit. Eicosanoid extracts were 12 times more concentrated compared with the original supernatants. The levels of 15(*S*)-HETE in the extracts were determined by using 15(*S*)-HETE enzyme immunoassay kit (Enzo Life Sciences, Lausen, Switzerland) as recommended in the product manual. All absorbance measurements were performed in duplicate.

##### siRNA Transfection

VSMCs were seeded at the density of 0.5 × 10^6^ cells/6-cm dishes in the morning. After 7 h, cells were then transfected with 20 nmol/liters of siRNA either against 12/15-LO or Stealth RNAi^TM^ siRNA Negative Control (Invitrogen) using Oligofectamine^TM^ Transfection reagent (Invitrogen) and left in Opti-MEM® reduced serum medium (Invitrogen) overnight. Opti-MEM was then replaced with the starvation medium to let cells recover from transfection complexes. 24 h after transfection, VSMCs were stimulated with PDGF for 5 min, trypsinized shortly thereafter and divided into two halves: one to be used for the determination of knockdown efficiency (real time-quantitative polymerase chain reaction (RT-qPCR)) and the other for the detection of phospho-STAT3 levels upon PDGF stimulation (Western blot, 10 μg/slot of proteins were loaded on the SDS-PAGE gel). 12/15-LO knock-down was accomplished with 2 different oligonucleotide sequences: siRNA-1, sense, 5′-AACUGGAUUUCUGUGAAGG-3′; antisense, 5′-CCUUCACAGAAAUCCAGUUGC-3′ (custom designed by Applied Biosystems, Carlsbad, CA) and siRNA-2, sense, 5′-CGGAUUUCUUCCUUCUGGA-3′, antisense, 5′-UCCAGAAGGAAGAAAUCCG-3′ (Sigma). More details for the siRNA knockdown of Nox4 in VSMCs, together with data showing the achieved transfection efficiency, have been described elsewhere (26).

##### Real Time-Quantitative Polymerase Chain Reaction

RNA isolation and subsequent cDNA synthesis were performed according to the instructions of the manufacturer, using the PeqGOLD Total RNA kit (Peqlab, Erlangen, Germany) and Superscript^TM^ First-strand Synthesis System (Invitrogen), respectively. The RT-qPCR was carried out using Light Cycler^TM^ LC480 SYBR Green I Master reagent (Roche Diagnostics) in reaction volume of 15 μl. QuantiTect® Primer Assay for rat leukocyte-type 12/15-LO was ordered from Qiagen (Düsseldorf, Germany, catalog number QT00181265), diluted, and aliquoted as recommended by the manufacturer. Forward and reverse primers for the housekeeping gene *18S* were from Invitrogen and their respective sequences were as follows: GAATTGACGGAAGGGCACCACCAG and GTGCAGCCCCGGACATCTAAGG. PCR contained one denaturation step (10 min at 95 °C) and up to 50 amplification cycles (annealing step, 30 s at 61 °C and elongation step, 15 s at 72 °C). Melting curves of the amplified DNA were analyzed to make sure that the PCR resulted in amplification of one specific product only. Data were analyzed using Light Cycler^TM^ LC480 software and the 2^−ΔΔ^*^Ct^* method.

##### Proliferation Assay in hAOSMC

hAOSMCs were seeded at the density of 15,000 cells/well in a 96-well plate (Microtest^TM^, Optilux^TM^, black/clear bottom, TC Surface, sterile with lid, BD Biosciences) to grow for the next 24 h and starved overnight. The starvation medium was freshly exchanged and cells were preincubated with either DMSO (0.1%) or increasing concentrations of I3MO (0.1–5 μm) for 30 min and stimulated with PDGF-BB (20 ng/ml) for the next 24 h. Cells were loaded with bromodeoxyuridine (BrdU, 10 μm) 4 h after PDGF stimulation. BrdU incorporation was measured according to the manufacturer's instructions using the BrdU Cell Proliferation ELISA kit (chemiluminescent) from Roche Diagnostics GmbH.

##### Statistics

Statistical analyses of experiments are based on one-way analysis of variance and Bonferroni's post-test of selected data sets (after log transformation when appropriate). The effect was considered significant when *p* value was less than 0.05. Statistics and graph illustrations were carried out in GraphPad Prism 5.0 (GraphPad Software, Inc., San Diego, CA). Graphs represent values of at least three independent experiments, mean ± S.D.

## RESULTS

### 

#### 

##### Involvement of ROS in PDGF-induced STAT3 Phosphorylation

**FIGURE 1.**
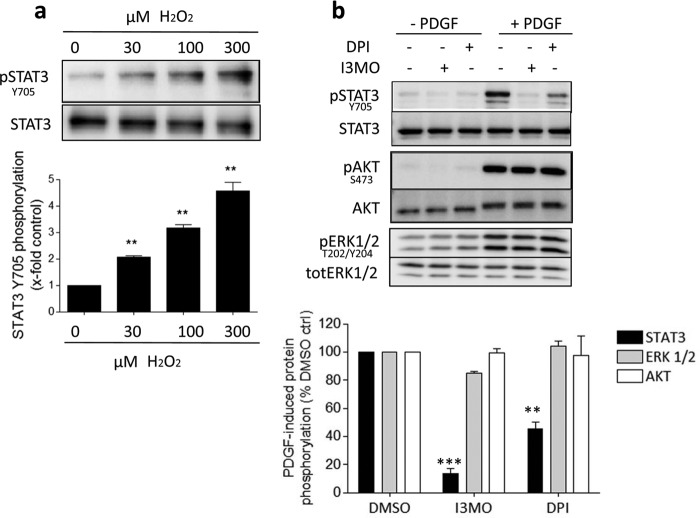
**STAT3 phosphorylation is redox-sensitive in VSMC.** *a*, serum-starved VSMCs were stimulated with H_2_O_2_ (0–300 μm) for 10 min as indicated. Western blot analysis was performed to determine the levels of phosphorylated STAT3 and total STAT3. One representative blot and a graph showing mean ± S.D. from densitometric analyses (expressed as fold PDGF-induced STAT3 phosphorylation) from 3 independent experiments are depicted; **, *p* < 0.01. *b* and *c,* VSMCs were preincubated with dimethyl sulfoxide (*DMSO*), I3MO (3 μm), or DPI (3 μm) for 30 min and stimulated with PDGF (20 ng/ml) for 10 min as indicated. The influence of the two compounds on the downstream PDGF signaling pathway was examined using Western blot. *b*, one representative blot, and *c*, a graph with mean ± S.D. from densitometric analyses (expressed as % PDGF-induced phosphorylation compared with DMSO-treated control cells) of 3 independent experiments are depicted (***, *p* < 0.001; **, *p* < 0.01).

H_2_O_2_ can serve as a messenger transducing receptor tyrosine kinase signals to downstream targets (23). To test whether STAT3 can be activated by ROS, we exposed VSMCs to exogenous H_2_O_2_ for 10 min, a time that leads to STAT3 activation in response to PDGF (1). H_2_O_2_ elicited a significant and concentration-dependent induction of STAT3 phosphorylation at Tyr^705^ (Fig. 1*a*).

We corroborated the redox control of PDGF-induced STAT3 phosphorylation in VSMCs in experiments with DPI. DPI acts as an unspecific inhibitor of flavoprotein-containing ROS producing enzymes, like NADPH oxidases or complexes I, III, and IV in the mitochondrial respiratory chain (MRC) (27, 28) and markedly blunts the intracellular ROS load. Remarkably, DPI-treated cells exhibited the same signaling pattern as those treated with I3MO upon PDGF stimulation: STAT3 phosphorylation at Tyr^705^ was inhibited without an effect on the phosphorylation and activation of Akt and ERK1/2 kinases (Fig. 1, *b* and *c*). These data are in concordance with other studies showing STAT3 activation as being highly redox sensitive (13–17). They, additionally, imply that I3MO might exhibit its effect on PDGF-induced STAT3 by interfering with a redox-regulated signaling pathway.

##### Role of Src Kinase for STAT3 Inhibition by I3MO

**FIGURE 2.**
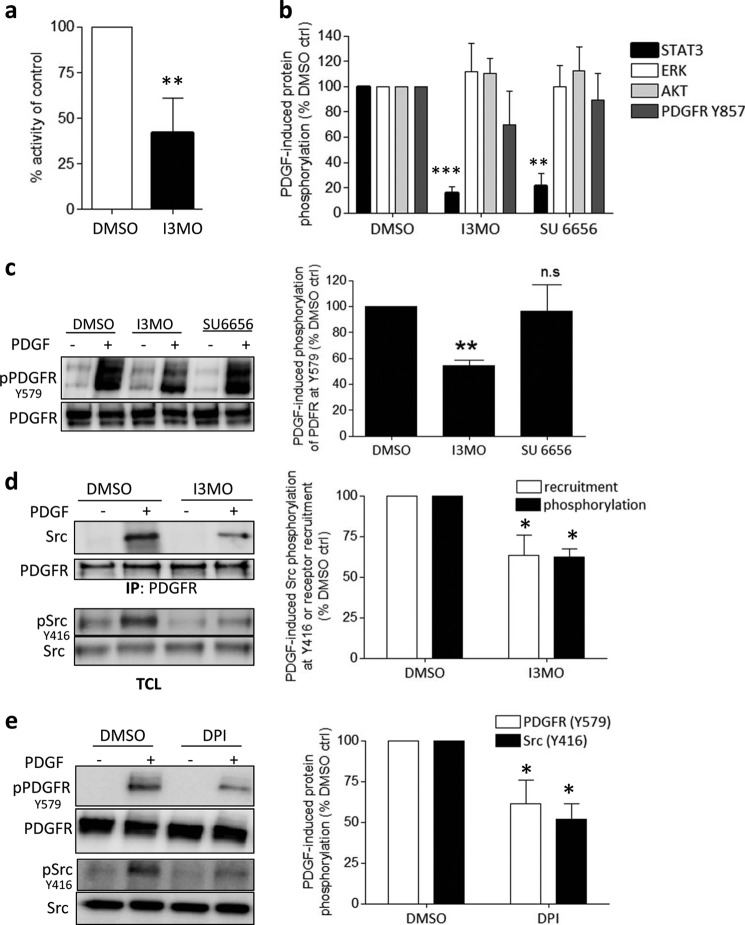
**Src is the upstream STAT3 kinase in PDGF-stimulated VSMC, and I3MO interferes with Src activity and activation.** *a,* results of an *in vitro* Src kinase activity assay, performed as described under ”Experimental Procedures.“ The *graph* shows mean ± S.D. of the percent inhibition from 6 performed experiments; **, *p* < 0.01. *b,* compiled normalized densitometric analysis of three independent immunoblot experiments comparing the effect of I3MO (3 μm) and SU6656 (2 μm) on PDGF-induced (20 ng/ml, 5 min) phosphorylation of STAT3, ERK1/2, AKT, and PDGFR (Y857) in VSMC. PDGF-induced phosphorylation in dimethyl sulfoxide (*DMSO*)-treated control cells was set as 100%. *Graph* depicts mean ± S.D.; ***, *p* < 0.001; **, *p* < 0.01). *c,* VSMC were serum-starved, pretreated with DMSO (0.1%), I3MO (3 μm), or SU6656 (2 μm) as indicated for 30 min before they were stimulated with PDGF (20 ng/ml) for 5 min. Total cell lysates were subjected to immunoblot analysis for phospho-PDGFR(Y579) and total PDGFR levels. Representative blots out of three independent experiments are depicted. The *graph on the right* depicts compiled densitometric data of all experiments. PDGF-induced phosphorylation in DMSO-treated control cells was hereby set as 100% (**, *p* < 0.01; mean ± S.D.). *d,* VSMC were serum starved, pretreated with DMSO (0.1%) or I3MO (3 μm) as indicated for 30 min before they were stimulated with PDGF (20 ng/ml) for 5 min, and lysed. 750 μg of protein extract was subjected to immunopreciptation with anti-PDGFR antibody. The immunoprecipitates were probed for Src and PDGFR (*IP*). An aliquot of the same lysates was subjected to immunoblot analysis for phospho-Src (Y416) and total Src (*TCL*). Representative blots of three experiments are depicted. The *graph on the right* depicts compiled and normalized data (PDGF-induced phosphorylation/recruitment in DMSO-treated cells was set 100%) of all performed experiments (*, *p* < 0.05, mean ± S.D.). *e*, VSMC were serum-starved, pretreated with DMSO (0.1%) or DPI (3 μm) as indicated for 30 min, stimulated with PDGF (20 ng/ml) for 5 min before total cell lysates were subjected to a immunoblot analysis for phospho-PDGFR(Y579), phospho-Src (Y416), PDGFR, and Src levels. Representative blots from three independent experiments with consistent results are depicted. The *graph on the right* depicts compiled and normalized data (PDGF-induced phosphorylation in DMSO control cells is set 100%) of all performed experiments (*, *p* < 0.05, mean ± S.D.).

Next, we investigated whether interference of I3MO with ROS-dependent STAT3 signaling involves Src kinase. Src is a redox-sensitive protein-tyrosine kinase capable of phosphorylating STAT3 (29) and proposed as direct target of some indirubin derivatives in cancer cells (30). In an *in vitro* assay, I3MO (3 μm) indeed inhibited Src kinase activity up to 50% (Fig. 2*a*). Moreover, SU6665, a known selective Src inhibitor (31), abolished PDGF-induced STAT3 phosphorylation in rat VSMC without significantly affecting phosphorylation of Akt, ERK, and PDGFR at the activation loop Tyr^857^, mirroring the profile of I3MO (Fig. 2*b*). A look at the phosphorylation of Tyr^579^ in the PDGFR, known to constitute the binding site for the Src homology 2 domain of Src kinases (32), uncovered that I3MO, in contrast to SU6656, interfered with this phosphorylation (Fig. 2*c*). In line with existing knowledge about early PDGFR signaling (33), Src was recruited to a lesser extent to the activated PDGFR and showed a reduced activating phosphorylation at Tyr^416^ in I3MO-treated VSMC compared with control cells (Fig. 2*d*). These data indicate that Src is a major kinase mediating STAT3 phosphorylation in PDGF-stimulated rat VSMC, and that I3MO may act as a direct Src kinase inhibitor. In contrast to mere Src kinase inhibitors, I3MO also interfered with Src activation at the upstream step of recruitment of the kinase to the activated PDGFR. Notably also, the ROS-reducing DPI hampered phosphorylation of PDGFR at Tyr^579^ and Src at Tyr^416^ (Fig. 2*e*). This underlines the essential role of ROS for the signal transduction, PDGFR → Src → STAT3, which we focused on in the following experiments.

##### Inhibition of PDGF-induced ROS Production by I3MO

**FIGURE 3.**
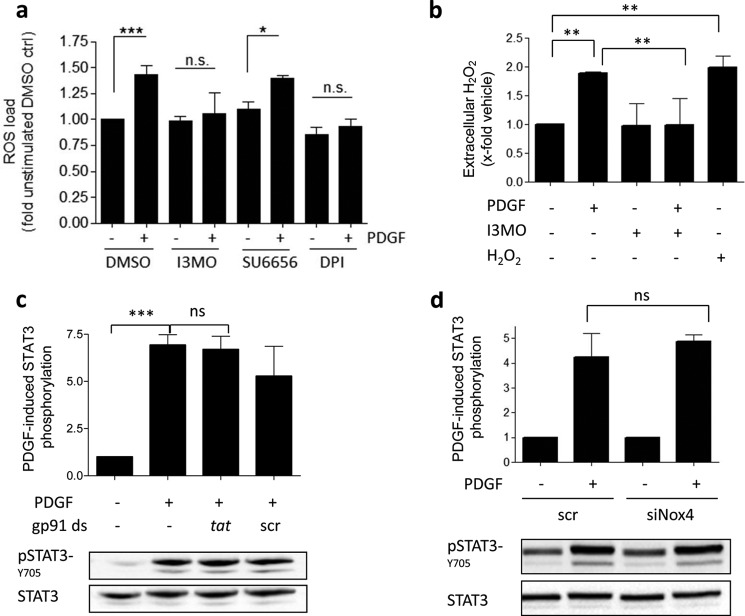
**I3MO inhibits PDGF-induced generation of ROS, and ROS involved in STAT3 phosphorylation do not originate from Nox1 or Nox4.** *a,* VSMCs were pre-treated with DMSO (0.1%), I3MO (3 μm), SU6656 (2 μm), or DPI (3 μm) for 30 min and stimulated with PDGF (20 ng/ml) for 10 min. A fluorescent probe, H_2_DCF-DA, was added to cells 25 min prior to the flow cytometric assessment of oxidized DCF. *Graph* shows relative mean ± S.D. of fluorescence intensity from a minimum of 3 independent experiments; *, *p* < 0.05; ***, *p* < 0.001; *ns*, not significant. *b,* VSMCs were preincubated with DMSO (−) or I3MO (5 μm) for 30 min and stimulated with PDGF (20 ng/ml) for an additional 30 min in Amplex® Red medium in the presence or absence of catalase. Incubation with H_2_O_2_ (0.5 μm) for 30 min served as a positive control. Supernatants were transferred into a 96-well plate for the fluorescence intensity measurement in triplicate. *Graph* shows relative mean ± S.D. of catalase-corrected fluorescence from 3 independent experiments; **, *p* < 0.01. *c,* VSMCs were pre-treated with a Nox1-specific inhibitory peptide, gp91ds-*tat* (100 μm) or with a scrambled control, gp91ds-scr (100 μm), for 30 min and stimulated with PDGF (20 ng/ml) for 10 min as indicated. Western blot was used to determine the effect of Nox1 inhibition on STAT3 phosphorylation. A graph with mean ± S.D. from densitometric analyses out of 3 independent experiments and one representative blot are shown; ***, *p* < 0.001. *d,* VSMCs were transiently transfected with either a silencing RNA directed against the Nox4 isozyme or a scrambled RNA control. At the time of most efficient Nox4 silencing, cells were stimulated with PDGF (20 ng/ml) for 10 min. Western blot was used to examine the effect of Nox4 silencing on PDGF-induced STAT3 phosphorylation. A *graph* with mean ± S.D. from densitometric analyses out of 4 independent experiments and one representative blot are shown.

We determined whether I3MO may directly interfere with ROS formation induced by PDGF in VSMCs. Using H_2_DCF-DA (34), we observed that PDGF led to a significant increase in intracellular ROS 10 min after stimulation. I3MO blunted this PDGF-induced ROS production, comparable with DPI. Of note, the Src inhibitor SU6656 failed to inhibit PDGF-triggered ROS formation, indicating that ROS reduction by I3MO is independent of Src inhibition (Fig. 3*a*). The inhibition of PDGF-induced ROS formation by I3MO was confirmed by reduced extracellular H_2_O_2_ release as evident by measurements using the fluorogenic substrate Amplex® Red (Fig. 3*b*). I3MO itself did not show radical scavenging or reducing properties in the presence of 2,2-diphenyl-1-picrylhydrazyl and 3-(4,5-dimethylthiazol-2-yl)-2,5-diphenyltetrazolium bromide, respectively (data not shown). Thus, I3MO influences the activity of cellular ROS sources or detoxification systems.

##### Potential Sources for ROS Production in VSMCs: Nox Proteins and the MRC

In the next step we aimed to delineate the endogenous ROS source, which is crucial for PDGF-induced STAT3 phosphorylation. Nox proteins are usually considered as the most important ROS-producing enzymes and regulators of PDGF-induced signal transduction in VSMCs (15, 20, 22, 27, 35). Nox1 and Nox4 are the only two isoforms expressed in rat VSMCs (36). Stimulus-dependent activation of Nox1 requires the recruitment of the cytosolic to its membrane-residing subunits. To decipher the role of Nox1 for PDGF-triggered STAT3 activation in our cells, we used the cell-permeable blocking peptide gp91ds-*tat* that competes with the membrane bound gp91*^phox^* for the cytosolic p47*^phox^* and thus prevents Nox1 membrane assembly (37). The functionality of this inhibitory peptide in our cell system has been shown previously (26). Here, we demonstrate that specific Nox1 inhibition does not affect PDGF-induced signaling toward STAT3 activation (Fig. 3*c*). In contrast to Nox1, Nox4 is considered to be constitutively active and is regulated on the transcriptional level (28, 37). We therefore used a siRNA approach to down-regulate Nox4, as described in detail in a previous study (26). PDGF stimulation of cells with depleted Nox4 levels did not result in reduced STAT3 phosphorylation (Fig. 3*d*). We conclude that neither Nox1 nor Nox4 are involved in PDGF-induced signal transduction toward STAT3 in rat VSMCs.

ROS molecules, like superoxide and H_2_O_2_, can result from an inefficient/leaky electron transfer process in the MRC (38), predominantly at complex I and complex III (39). Mitochondria are sensors, as well as sources of oxidative stress and were described to be important in the development of atherosclerosis (40). To examine a possible role of mitochondrial ROS for PDGF-induced STAT3 phosphorylation in VSMC, we used several compounds that interfere with MRC-derived ROS formation and assessed their impact on STAT3 activation. Neither the mitochondrial antioxidant mitoquinone (MitoQ), nor the mitochondrial uncoupling agent, carbonyl cyanide *m*-chlorophenyl hydrazone affected STAT3 phosphorylation upon 10 min PDGF stimulation (see Table 1). In contrast, the complex I inhibitor, rotenone showed an inhibitory effect on STAT3, although not concentration dependently and, thus, very likely unspecifically. We therefore excluded mitochondrial ROS as involved players in the PDGFR-STAT3 signaling axis.

**TABLE 1:** **Investigation of inhibitors of different ROS sources for a selective inhibition of PDGF-induced STAT3 Tyr^705^ phosphorylation**

Inhibitor	Concentration	Action	Inhibition of PDGF-induced STAT3 phosphorylation	Inhibiting PDGF-induced Akt, ERK1/2, and Src activation
DPI	3 μm	Flavoprotein inhibitor	Yes	No
*N*-Acetylcystein	2 mm	Glutathione precursor	Yes	No
Apocynin	1 mm	Nox inhibitor (unspecific)	No	
gp91-*tat*	100 μm	Nox1*^a^* inhibitory peptide	No	
Rotenone	0.5–10 μm	Complex I inhibitor	Yes, but without dose response	No
Carbonyl cyanide *m*-chlorophenyl hydrazone	250 nm	Mitochondrial uncoupler	No	
Mitoquinone	200 nm	Mitochondrial antioxidant	No	
Nordihydroguaia-retic acid (NDGA)	5–100 μm	Lipoxygenase inhibitor	Yes	No
Acetylsalycilic acid	100 μm	COX*^b^* inhibitor	No	
Allopurinol	250 μm	Xanthine oxydase inhibitor	No	
l-Nitroarginine methyl ester (l-NAME)	200 μm	NOS*^c^* inhibitor	No	

*^a^* NADPH oxidase.

*^b^* Cyclooxygenase.

*^c^* Nitric-oxide synthase.

##### From PDGF Receptor to STAT3 Phosphorylation: Role of 12/15-Lipoxygenase

**FIGURE 4.**
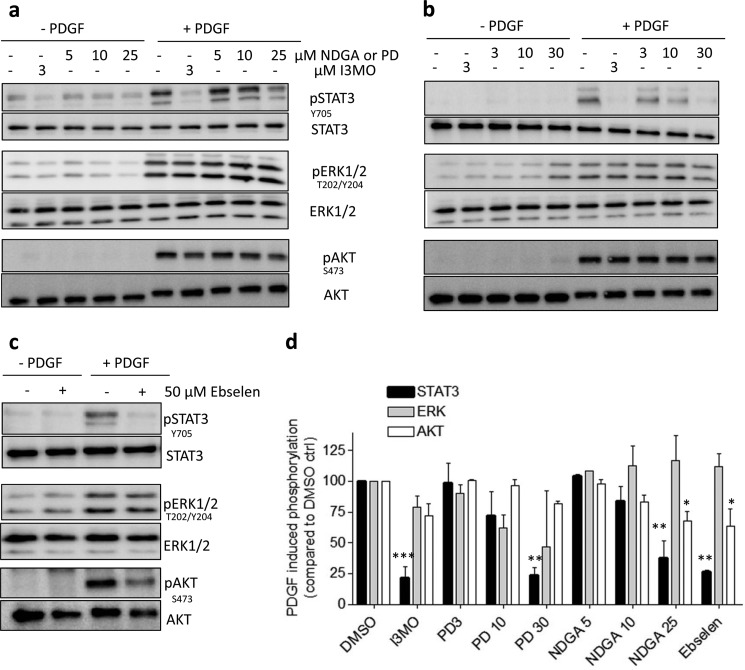
**Inhibitors of 12/15-LO mimic the selective effect of I3MO on STAT3 phosphorylation.** VSMCs were treated with dimethyl sulfoxide (*DMSO*) (−) or the indicated concentrations of I3MO, (*a*) NDGA, a pan-LO inhibitor, (*b*) PD 146176 (*PD*), or (*c*) ebselen, more selective 12/15-LO inhibitors, for 30 min and subsequently stimulated with PDGF (20 ng/ml) for 10 min. Western blot was used to determine phosphorylation and total levels of STAT3, AKT, and ERK1/2. *d,* the overview graph depicts compiled and normalized densitometric values from 3 independent experiments. The PDGF-induced phosphorylation of the respective protein in DMSO-treated control cells was set 100%. *, *p* < 0.05; **, *p* < 0.01; mean ± S.D.

**FIGURE 5.**
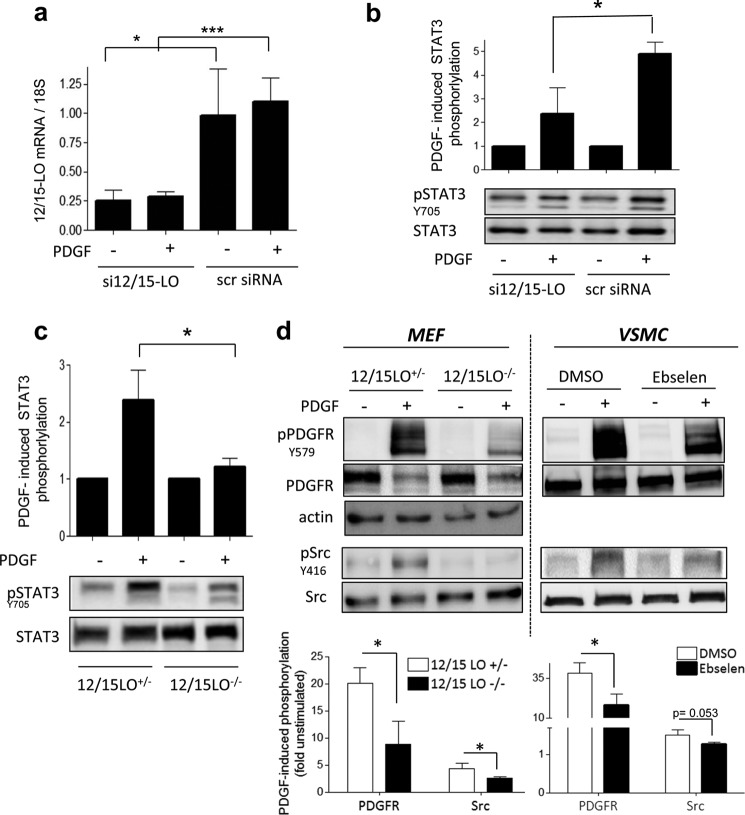
**Reduced 12/15-LO expression or activity impairs the PDGF-induced PDGFR(Y579), Src(Y416), and STAT3(Y705) phosphorylation.** *a* and *b,* VSMCs were transfected with siRNA targeting 12/15-LO (*si12*/*15-LO*) or scrambled control (*scr siRNA*). 24 h after transfection, cells were stimulated with PDGF (20 ng/ml) for 5 min. They were then detached using trypsin/EDTA solution and divided into two halves, one to be used for the mRNA extraction and subsequent RT-qPCR to determine the knockdown efficiency (*a*), and other for the Western blot determination of STAT3 phosphorylation (*b*). *a, graph* shows relative mean ± S.D. of 12/15-LO mRNA levels (normalized to 18S) from at least 4 independent experiments. *b,* a *graph* with mean ± S.D. of STAT3 phosphorylation assessed by densitometric analyses of 4 experiments and one representative blot are shown. *, *p* < 0.05; *ns*, not significant. *c*, STAT3 phosphorylation was examined after 5-min PDGF (20 ng/ml) stimulations in heterozygous 12/15-LO^+/−^ and homozygous 12/15-LO^−/−^ MEFs using Western blot. The graph shows relative mean ± S.D. of densitometric analyses out of 3 experiments; *, *p* < 0.05. One representative blot is shown. *d,* PDGF-induced phosphorylation of Src(Y416) and PDGFR(Y579) was examined after a 5-min PDGF (20 ng/ml) stimulation in heterozygous 12/15-LO^+/−^ and homozygous 12/15-LO^−/−^ MEFs (*left panel*) as well as DMSO or ebselen-(50 μm)-treated VSMC (*right panel*) using Western blot. Representative blots are depicted. The *bar graphs* show compiled densitometric analyses of all performed experiments (mean ± S.D., expressed as fold PDGF-induced phosphorylation (compared with quiescent cells), *, *p* < 0.05).

To pin down the source of ROS responsible for STAT3 activation, we administered commonly used inhibitors for additional different ROS sources in VSMCs and checked for reduction of PDGF-induced ROS (data not shown) and their (selective) inhibitory action on STAT3 over ERK1/2 and AKT. With this approach, we excluded a potential role of nitric-oxide synthases, cyclooxygenases, and xanthine oxidase in PDGF-induced STAT3 phosphorylation (Table 1). However, when VSMCs were preincubated with NDGA, a pan-lipoxygenase (LO) inhibitor, both PDGF-induced ROS (not shown) and STAT3 activation were completely blocked by 25 μm NDGA (Fig. 4, *a* and *d*). Additionally, PDGF-induced phosphorylation of Akt and ERK1/2 remained intact or was only weakly affected (Fig. 4, *a* and *d*), resembling the signaling pattern of PDGF-activated VSMCs treated with I3MO. Mammalian LO are non-heme iron-containing dioxygenases that insert molecular oxygen into polyunsaturated fatty acids, like arachidonic or linoleic acid (41). According to the C-atom of the arachidonic acid where the oxygen is incorporated, this class of enzymes is subdivided in mammals into 5-, 8-, 12-, and 15-LOs (42). Further differentiation of LO enzymes is based on genetic and biochemical data. For example, a murine leukocyte-type 12-LO shares a relatively high degree of homology with human and rabbit reticulocyte-type 15-LO. Using arachidonic acid as substrate both enzymes can produce 12(*S*)- and 15(*S*)-HpETE (which are usually rapidly metabolized to 12(*S*)- and 15(*S*)-HETE) and are therefore termed as 12/15-LO. Although LOs are a group of enzymes comprising several different subtypes (42), the leukocyte-type 12/15-LO was most often described to be present and to play a role in signal transduction in VSMCs (41, 43). We, therefore, examined more selective 12/15-LO inhibitors for their influence on PDGF-induced STAT3 activation. The 12/15-LO inhibitor PD 146176 blocked the PDGF-induced STAT3 phosphorylation at 30 μm, without negatively affecting the activation of Akt and ERK1/2 (Fig. 4, *b* and *d*). Ebselen, a glutathione peroxidase mimetic selenoorganic compound and described to mainly counteract 12/15-LO signaling (44), also showed a strong inhibitory effect on STAT3 phosphorylation, a weaker inhibition toward Akt phosphorylation and no inhibition toward ERK1/2 (Fig. 4, *c* and *d*). However, the rather high concentration of PD 146176 (30 μm) may not guarantee truly specific inhibition of 12/15-LO, and ebselen has recently been reported to inhibit Nox1-mediated ROS production as well (45). We therefore corroborated the 12/15-LO dependence of the PDGF-mediated signaling toward STAT3 by using a targeted siRNA approach against leukocyte-type 12/15-LO in VSMC. The maximum silencing (approximately 70%) of 12/15-LO was achieved 24 h after transfection (Fig. 5*a*). Cells with silenced 12/15-LO and stimulated with PDGF showed an impaired STAT3 phosphorylation compared with cells transfected with the scrambled control (Fig. 5*b*). Furthermore, and in accordance with our data in VSMC, we were able to observe stronger STAT3 activation with increasing expression levels of leukocyte 12-LO, the murine representative of the 12/15-LO family, in embryonic fibroblasts (Fig. 5*c*). These findings underline an important role of 12/15-LO activity for STAT3 activation that may even be true for cell types other than VSMCs upon PDGF stimulation. In line with our observations in Fig. 2, interference with 12/15-LO signaling, either by genetic knockdown of the enzyme in MEF or by ebselen in VSMC, also impaired phosphorylation of the PDGFR at Tyr^579^ and phosphorylation of Src at Tyr^416^ (Fig. 5*d*). Total PDGFR levels were reduced in MEF after a 5-min PDGF exposure, which is most likely due to rapid receptor internalization and degradation of the receptor in this cell type (but not due to uneven protein loading as seen by the actin blot). Ebselen reproducibly modestly reduced PDGF-induced Src phosphorylation, almost reaching significance after three performed experiments (*p* = 0.053).

**FIGURE 6.**
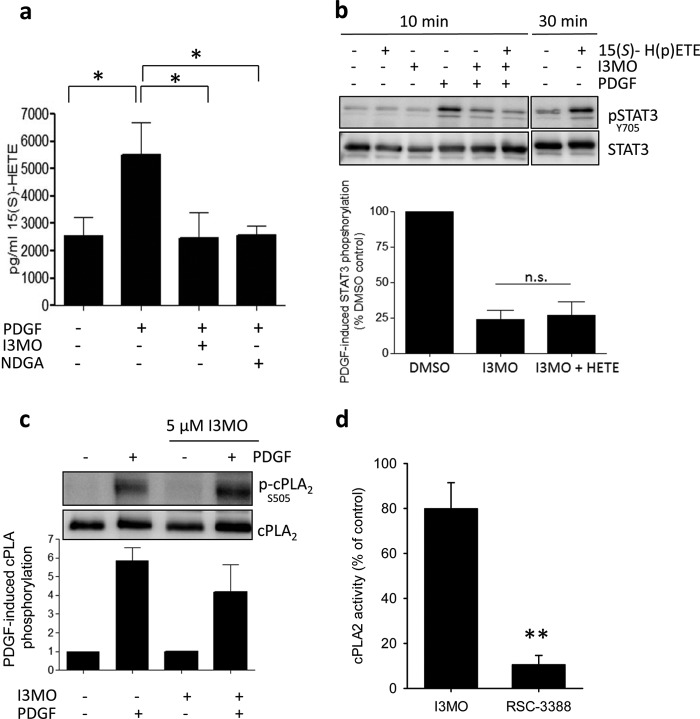
**I3MO inhibits the PDGF-induced 15(*S*)-HETE production without affecting phosphorylation and enzyme activity of cPLA2, and exogenous 15(*S*)-H(p)ETE cannot rescue STAT3 phosphorylation in I3MO-treated cells.** *a,* VSMCs were preincubated with dimethyl sulfoxide (*DMSO*) (−), I3MO (5 μm), or NDGA (20 μm) for 30 min and stimulated with PDGF (20 ng/ml). Supernatants were taken 10 min after PDGF stimulation for the extraction of eicosanoids and the determination of 15(*S*)-HETE levels in an enzyme immunoassay. *Graph* shows mean ± S.D. out of 4 to 5 experiments, all measured in duplicate; *, *p* < 0.05. *b*, quiescent VSMC were pretreated with I3MO (3 μm) for 30 min and then stimulated with PDGF (20 ng/ml) and/or 15(*S*)-H(p)ETE (1 μm; 1:1 mixture of 15(*S*)-HETE: 15(*S*)-HpETE) for 10 min. Total cell lysates were subjected to Western blot analysis for pSTAT3 (Y705) and total STAT3. Representative blots out of 3 independent experiments with consistent results are depicted. To ensure functionality of the 15(*S*)-H(p)ETE mixture, quiescent VSMC were stimulated with 15(*S*)-H(p)ETE for 30 min, which induced marked STAT3 phosphorylation as reported previously for 15(*S*)-HETE by others (50, 52). The *lower bar graph* depicts PDGF-induced phosphorylation of STAT3 (after 10 min), compiled from all performed experiments and expressed as % phosphorylation of DMSO control cells. (mean ± S.D.). *c*, VSMCs were pretreated with DMSO (−) or I3MO (5 μm) for 30 min and stimulated with PDGF (20 ng/ml of PDGF) for 10 min. Western blot was used to examine levels of total cPLA_2_ and cPLA_2_ phosphorylated at Ser^505^. One representative blot of 3 independent experiments is shown. The *bar graph* below depicts compiled densitometric data showing PDGF-induced phosphorylation. *d,* isolated cPLA_2_ was pretreated with DMSO, I3MO (10 μm), or a control inhibitor RSC-3388 (0.1 μm) for 15 min at 37 °C prior to addition of phospholipid substrate 1-palmitoyl-2-arachidonyl-*sn*-glycero-3-phosphocholine and 1-palmitoyl-2-oleoyl-*sn*-glycerol. *Graph* shows mean ± S.D. of the cPLA_2_ activity relative to vehicle control out of 3 experiments (**, *p* < 0.01).

The next obvious question to answer then was whether I3MO interferes with 12/15-LO. Porcine aortic smooth muscle cells were shown to display elevated 12/15-LO products after PDGF stimulation (46). We, thus, examined whether this holds true for rat VSMCs and also whether I3MO could reduce 12/15-LO product formation. I3MO and the positive control NDGA indeed blocked the production of the 12/15-LO product 15(*S*)-HETE, which was significantly elevated within 10 min of PDGF stimulation, as quantified by an enzyme immunoassay (Fig. 6*a*). The decrease of PDGF-induced STAT3 phosphorylation by I3MO may, thus, in turn be a result of reduced 12/15-LO activity. Of note, addition of 15(*S*)-H(p)ETE was not able to rescue STAT3 phosphorylation in I3MO-treated VSMC within 10 min of PDGF stimulation in our cell system. This may suggest that another 12/15-LO-derived (by)product mediates the early signal transduction from PDGF to STAT3 (Fig. 6*b*).

I3MO did not inhibit purified human 12/15-LO in an *in vitro* enzyme assay (data not shown). Also, I3MO failed to interfere with the supply of arachidonic acid, the substrate for 12/15-LO. Neither PDGF-induced phosphorylation/activation of cytosolic phospholipase A_2_ (cPLA_2_), that causes release of arachidonic acid from membrane lipids, nor the activity of the purified cPLA_2_ were markedly influenced by I3MO at concentrations used in our experiments (Fig. 6, *c* and *d*).

##### The Relevance of 12/15-LO in PDGF-induced Signaling toward STAT3 in hAOSMCs

**FIGURE 7.**
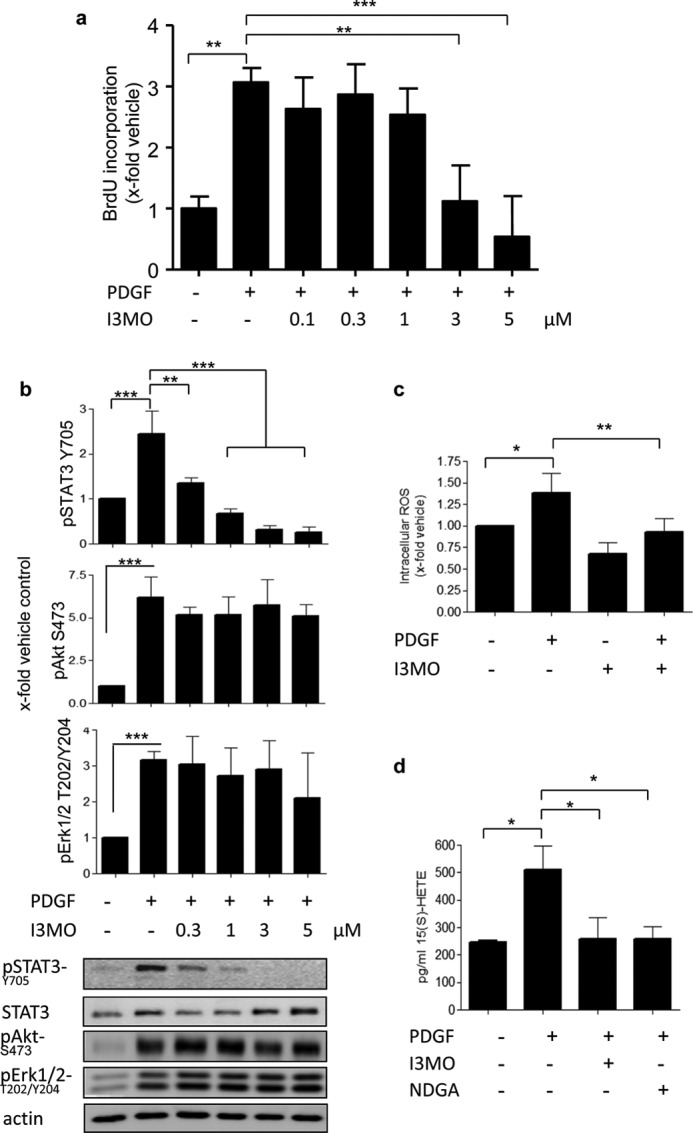
**Inhibition of PDGF-induced cell proliferation, STAT3 phosphorylation, intracellular ROS formation, and 15(*S*)-HETE production in hAOSMCs by I3MO.** *a,* results of the BrdU incorporation assay after a 24-h PDGF stimulation. A *graph* shows the relative mean ± S.D. out of 3 experiments, each measured in triplicates; **, *p* < 0.01; ***, *p* < 0.001. *b,* Western blot analysis of PDGF-induced pSTAT3, pAkt, and pERK1/2 in the presence of increasing concentrations of I3MO. One representative blot and graphs showing mean ± S.D. of densitometric analyses of 3 independent experiments are depicted: *, *p* < 0.05; **, *p* < 0.01; ***, *p* < 0.001; *ns*, not significant. *c,* influence of I3MO (5 μm) on the PDGF-induced intracellular ROS formation, determined using a fluorescent probe, H_2_DCF-DA. *Graph* shows relative mean ± S.D. of DCF fluorescence out of 4 independent experiments; *, *p* < 0.05; **, *p* < 0.01. *d,* the effect of I3MO (5 μm) and NDGA (20 μm) on PDGF-induced 15(*S*)-HETE production, examined in the corresponding enzyme immunoassay. *Graph* represents mean ± S.D. out of 3 experiments; *, *p* < 0.05.

To examine whether 12/15-LO is also required for PDGF-induced STAT3 phosphorylation in human cells, we repeated key experiments with I3MO in hAOSMCs. I3MO inhibited PDGF-induced proliferation and PDGF-induced STAT3 phosphorylation concentration dependently, the latter with a significant effect at already 0.3 μm (Fig. 7, *a* and *b*). The activation of Akt and ERK1/2 kinases was not affected by I3MO in hAOSMCs either (Fig. 7*b*). I3MO abolished the PDGF-induced intracellular ROS formation as measured by using H_2_DCF-DA and successfully blocked 15(*S*)-HETE production, as evident in the respective enzyme immunoassay (Fig. 7, *c* and *d*). These results show that I3MO successfully inhibited cellular 15-LO product synthesis, the corresponding human isoform of 12/15-LO also in hAOSMCs. Furthermore, the results are in line with the notion that PDGF-induced STAT3 phosphorylation in hAOSMCs might require the activity of 15-LO.

## DISCUSSION

Starting off with I3MO as a selective small molecule inhibitor of STAT3 in VSMCs we show here that (i) PDGF-induced signaling toward STAT3 in VSMCs requires ROS and (ii) that this ROS-dependent signaling pathway from the PDGF-receptor to STAT3 in VSMCs relies on 12/15-LO, which mediates optimal recruitment of Src to the activated receptor. We show for the first time that I3MO interferes with 12/15-LO activity in PDGF-stimulated VSMCs, blunts ROS and 15(*S*)-HETE production, and does not only directly inhibit the kinase activity but also the activation of Src by PDGF.

The important role of 12/15-LO in PDGF-mediated superoxide production in VSMCs was previously suggested (47), and a recent study reported that 12/15-LO-derived lipid peroxides participate in oxidative inactivation of protein-tyrosine phosphatases controlling PDGF receptor signaling (48). As protein-tyrosine phosphatases show a certain degree of site specificity in their action (49), impaired 12/15-LO activity and subsequently increased protein-tyrosine phosphatase activity could explain the reduced Tyr^579^ phosphorylation of PDGFR in I3MO-treated VSMC with concomitant unaltered Tyr^857^ phosphorylation in this study. Our findings are the first to directly link LO activity and STAT3 activation during PDGF signaling. They complement studies using the LO product 15(*S*)-HETE as a direct stimulus and observing late STAT3 phosphorylation (>15–30 min) in vascular smooth muscle and endothelial cells (50–52). The redox-regulated signaling axis PDGF → 12/15-LO → phosphorylation of PDGFR at Tyr^579^ → Src recruitment → STAT3 activation, however, is revealed for the first time in this study.

12/15-LO is dependent on the redox status of its close environment, because the active enzyme requires the oxidation of the ferrous ion in its catalytic center into a trivalent ferric ion (53). This may explain why DPI and general antioxidants such as *N*-acetylcysteine, although not directly acting as 12/15-LO inhibitors, selectively interfere with STAT3 phosphorylation in PDGF-stimulated VSMC, and why H_2_O_2_ induces STAT3 phosphorylation (see Fig. 1*b* and Table 1). We have not revealed the actual target of I3MO responsible for inhibition of 12/15-LO signaling, but strongly assume interference of I3MO with an event upstream of 12/15-LO activation in VSMCs. We have excluded radical scavenging, direct inhibition of 12/15-LO, and impaired activity or activation of cPLA_2_ by I3MO.

We were able to rule out common ROS sources, *i.e.* mitochondria as well as Nox1 and -4, to be involved in STAT3 phosphorylation upon PDGF stimulation in rat VSMC. Human vascular smooth muscle cells also express the Nox5 isoform, and one study suggests a role of Nox5 in PDGF-induced human AOSMC proliferation implicating the Jak-STAT3 signaling pathway (15). Although we did not address specifically this issue and rather find Src as the main kinase upstream of STAT3, the obtained data with I3MO in hAOSMCs, which are highly comparable with those in rat cells, suggest that Nox5 may not be the only source of ROS involved in signal transduction from PDGF to STAT3 in human VSMC.

Overall, our study shows for the first time that I3MO inhibits PDGF-induced ROS and 15(*S*)-HETE formation in PDGF-activated VSMCs, and this effect could be linked to the previously reported inhibition of STAT3. More importantly, we unravel the importance of 12/15-LO activity for the PDGF-induced and Src-mediated STAT3 activation in rat VSMC.

## Footnotes

2 The abbreviations used are:

PDGF(R): platelet-derived growth factor (receptor)
12/15-LO: 12/15-lipoxygenase
cPLA_2_: cytosolic phospholipase A_2_
DPI: diphenyleneiodonium
H_2_DCF-DA: 2′,7′-dihydrodichlorofluorescein-diacetate
hAOSMC: primary human aortic smooth muscle cells
HETE: hydroxyeicosatetraenoic acid
HpETE: hydroperoxyeicosatetraenoic acid
I3MO: indirubin-3′-monoxime
LO: lipoxygenase
MEF: mouse embryonic fibroblast
MRC: mitochondrial respiratory chain
NDGA: nordihydroguaiaretic acid
Nox: NADPH oxidase
ROS: reactive oxygen species
RT-qPCR: real time-quantitative polymerase chain reaction
VSMC: vascular smooth muscle cells.
